# Minimally disruptive medicine (MDM) in clinical practice: a qualitative case study of the human immunodeficiency virus (HIV) clinic care model

**DOI:** 10.1186/s12913-020-06010-x

**Published:** 2021-01-06

**Authors:** Abd Moain Abu Dabrh, Kasey R. Boehmer, Nathan Shippee, Stacey A. Rizza, Adam I. Perlman, Sara R. Dick, Emma M. Behnken, Victor M. Montori

**Affiliations:** 1 Department of Family Medicine, Mayo Clinic Florida, 4500 San Pablo Rd S, Jacksonville, FL USA; 2 Knowledge and Evaluation Research (KER) Unit, Mayo Clinic, Rochester, MN USA; 3 Integrative Medicine and Health, Division of General Internal Medicine, Mayo Clinic Florida, Jacksonville, FL USA; 4 Division of Health Policy and Management, University of Minnesota, Minneapolis, MN USA; 5 Division of Infectious Diseases, Mayo Clinic, Rochester, MN USA

**Keywords:** Minimally disruptive medicine, MDM, Cumulative complexity model, Healthcare burden, HIV care, Model of care, Capacity, Workload, Treatment burden, PILLARS

## Abstract

**Background:**

Recent evidence suggests the need to reframe healthcare delivery for patients with chronic conditions, with emphasis on minimizing healthcare footprint/workload on patients, caregivers, clinicians and health systems through the proposed Minimally Disruptive Medicine (MDM) care model named. HIV care models have evolved to further focus on understanding barriers and facilitators to care delivery while improving patient-centered outcomes (e.g., disease progression, adherence, access, quality of life). It is hypothesized that these models may provide an example of MDM care model in clinic practice. Therefore, this study aimed to observe and ascertain MDM-concordant and discordant elements that may exist within a tertiary-setting HIV clinic care model for patients living with HIV or AIDS (PLWHA). We also aimed to identify lessons learned from this setting to inform improving the feasibility and usefulness of MDM care model.

**Methods:**

This qualitative case study occurred in multidisciplinary HIV comprehensive-care clinic within an urban tertiary-medical center. Participants included Adult PLWHA and informal caregivers (e.g. family/friends) attending the clinic for regular appointments were recruited. All clinic staff were eligible for recruitment. Measurements included; semi-guided interviews with patients, caregivers, or both; semi-guided interviews with varied clinicians (individually); and direct observations of clinical encounters (patient-clinicians), as well as staff daily operations in 2015–2017. The qualitative-data synthesis used iterative, mainly inductive thematic coding.

**Results:**

Researcher interviews and observations data included 28 patients, 5 caregivers, and 14 care-team members. With few exceptions, the clinic care model elements aligned closely to the MDM model of care through supporting patient capacity/abilities (with some patients receiving minimal social support and limited assistance with reframing their biography) and minimizing workload/demands (with some patients challenged by the clinic hours of operation).

**Conclusions:**

The studied HIV clinic incorporated many of the MDM tenants, contributing to its validation, and informing gaps in knowledge. While these findings may support the design and implementation of care that is both minimally disruptive and maximally supportive, the impact of MDM on patient-important outcomes and different care settings require further studying.

**Supplementary Information:**

The online version contains supplementary material available at 10.1186/s12913-020-06010-x.

## Background

**Fig. 1:**
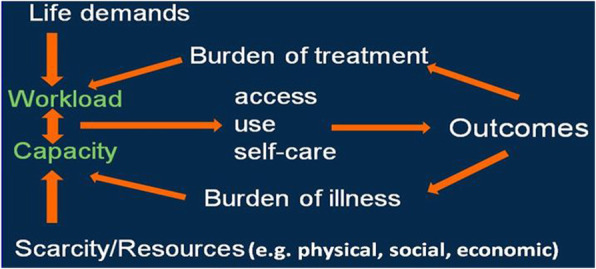
Cumulative Complexity Model (CuCoM)

In 2009, May, Montori and Mair proposed Minimally Disruptive Medicine (MDM), [1, 2] a paradigm shift in caring for those with chronic conditions. The proposal called for changing how health care is organized and delivered to reduce the burden of treatment for patients and caregivers, especially in light of their limited capacity to shoulder it. MDM proposed clinician-guided, evidence-based supportive care that takes into consideration the preferences and needs of the patients to provide care that fits into their context of daily lives [2]; thus minimizing the disruption or imbalance due to the added healthcare. Subsequently, MDM encompassed the grounded theory work of the Cumulative Complexity Model or CuCoM (Fig. 1) that explored elements that reflect and outline how these imbalances between patient *workload* and patient *capacity* affect care and outcomes [3].

Workload encompasses the demands on the patient’s time and energy, including demands of treatment, self-care, and life in general. Capacity encompasses the individual’s ability to handle work (e.g., personal, functional morbidity, financial/social resources, literacy) [4]. Workload-capacity imbalances reflect the effect of cumulative complexity; more workload and/or decreased/threatened capacity may lead to diminished self-care -enactment, access to care, and/or use of healthcare resources/adherence to plans; thus affecting patients clinical and QoL outcomes [5, 6]. Alternatively, supporting capacity may help overwhelmed patients experience better outcomes [7]. The evidence-based work of CuCoM supported the framework of MDM care model as one that pursues patient goals while minimizing the footprint/impact of healthcare with attention to capacity and workload, which is particularly important for patients with complex or chronic conditions [8, 9].

One condition requiring frequent, high-quality care is human immunodeficiency virus, or HIV. In particular, high adherence to antiretroviral therapy helps prevent complications and achieve near-normal life expectancy in People Living with HIV or AIDS (PLWHA) [10–15]. Stigma, fear and anxiety; poor access or experience with care; poorer mental health; unmet basic needs; inadequate transportation; and low health literacy – factors that limit patient capacity – are associated with reduced adherence to therapy [16–18]. A major threat to the wellbeing of PLWHA is “decreased desire and motivation to maintain vigilance in adhering to a treatment regimen among patient’s prescribed long-term protocols.” This so-called treatment fatigue maps closely to the concept of treatment burden under long-term management of a heavy workload of care [19–21]. Indeed, Claborn et al. have identified the pertinence of the CuCoM’s elements to HIV care [19, 21].

Models of HIV care – the HIV Care Continuum [22]— have focused heavily on improving outcomes (e.g., viral load, disease progression, mortality, quality of life) by facilitating access to well-coordinated, good quality care and promoting adherence in ways that fit into patients’ lives, reflecting in practice an implicit awareness of the CuCoM’s implications (Fig. 1). Although these care models have evolved without drawing directly from MDM, we hypothesize that clinics designed to care for PLWHA may have organically enacted many principles of MDM, including attention to patient workload-capacity balance, reduced treatment burden, and a focus on patient goals; thus, their success represents empirical evidence for MDM. Furthermore, we hypothesized that a thorough appreciation of the HIV clinic may contribute to improving the feasibility and usefulness of the MDM model. To understand the extent to which these hypotheses were valid, we conducted a qualitative case study of a well-established clinic designed for caring for PLWHA.

## Methods

The reporting of these results followed the Standards for Reporting Qualitative Research (SRQR) synthesis recommendations [23].

### Ethics approval and consent to participate

All study protocol and procedures were reviewed and approved by the Mayo Clinic Institutional Review Board (IRB) (study # 15–001876). The study was deemed as “minimal risk”. All participants in this study met with a research team member and received written and oral information; it included explaining the study intent, their participation in the study, and the complete autonomy to volunteer and participate in this study as well as their ability to withdraw from it without questions asked and at any time. Participants were given ample time to ask, discuss, and reflect on any questions before providing their consent to participate. Participating patients provided written consent while participating caregivers and clinicians provided oral consent. All participants received copy of the consent forms, including institutional and investigator’s contact information for any future communication or inquiry. All consent forms, consenting approaches, and study protocols were thoroughly reviewed and approved by the Mayo Clinic IRB.

### Study design

We conducted a qualitative case study of a multidisciplinary HIV comprehensive care practice embedded within a tertiary medical center in an urban setting in the United States. This design is suited to understand phenomena in real-life contexts, such as the study of a model of care for PLWHA [24]. Clinic staff used an IRB-approved recruitment flyer to inform patients and caregivers during a clinic visit about this study, and to assess their interest in participating. Sampling was consecutive while patients were coming to their regular appointments; after seeing the flyers and expressing interest; or after initial discussions with the clinicians.

Using semi-guided interviews (Supplementary Text File 1), we interviewed PLWHA receiving their care at this clinic with or without their informal caregivers (i.e. family members or friends providing support and/or care without financial reimbursement). The patients were given the option to be interviewed with or without the caregivers. In tandem, the caregivers were also asked for their permission and given the option to attend the interview, to participate in the interview, and/or to do neither. We also approached any clinician providing care to patients at this clinic to be interviewed separately and individually. All interviews were video- or audio-recorded (based on the participants’ preference). All participants were informed about the potential length of interviews and were given complete autonomy of when to stop the interview for any reason as well as scheduling it at their convenience. In addition, we directly observed daily-care operations and staff meetings and clinical-precepting interactions. Various patient-clinician encounters were observed and also video- or audio-recorded (based on preference). While the participants were not involved in the design, or conduct, or reporting, or dissemination plans of our research, many patients have extended their interest in reading about the published research, and we intend sharing these findings with them once they are formally published.

### The studied HIV care clinic

The clinic uses a team-coordinated approach to the care of PLWHA. Seven HIV-care consultant physicians and six specialty physicians in training (clinical fellows; Postgraduate years/PGY: PGY4, 5, and 6) work with two HIV-certified nurses in their primary care, the latter responsible for coordinating care, including arranging specialty referrals, completing preventive care, and educating patients. There are two on-site HIV pharmacists that counsel and educate patients on medication adherence, regimens, side effects, and drug interactions for HIV care and any other existing conditions. Three social workers and case managers assess and address educational, social, mental, legal, insurance, and financial needs, and provide support and counseling for patients as well as families and significant others, when disclosed.

### Participants

i. *Patients and caregivers*: Adult patients of the clinic and their caregivers (i.e., individuals attending appointments with or serving in a caregiving capacity for enrolled patients (e.g. family members, spouses, friends, and domestic partners). The exclusion criteria included participants unable to communicate clearly in English, legally blind, hearing impaired, clinically in unstable condition to engage in or provide consent for any other reason, or were coming for their first visit at the clinic.
ii. *Clinicians and other allied health professionals*: We recruited clinicians during monthly staff meetings or individually. Privately, a researcher approached each interested patient, discussed the study, and obtained the participant’s consent, including the potential of following up.

### Data collection

Data collected included the video- or audio-recording of the semi-guided interviews with participants, and the patient-clinician encounters (with or without caregivers in attendance) (as illustrated in Supplementary Figure 1) for subsequent analysis. We also collected data filed from written notes by research team member (AMAD) while directly observing daily-care operations and staff meetings and clinical-precepting interactions. All collected data were saved safely, and securely (locked cabinets for paper data; password and firewall-secured servers for digital data), with access only granted to the research team members. All recorded data were transcribed professionally and securely (i.e. maintain participant’ privacy and confidentiality by de-identifying and removing any potential personal identifiers).

### Data analysis

#### Transcribed participants interviews and clinical encounters

**Fig. 2:**
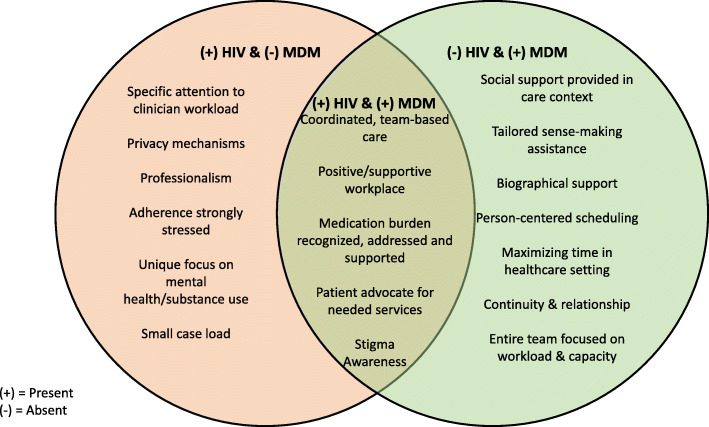
Concordant, discordant, or unique elements compared between MDM and the studied HIV Clinic Care Model

Data analysis consisted of iterative, mainly inductive coding cycles using theorizing approach that examined the general relationship between the identified categories of the data as described previously [25]. Source documents – transcripts and observation notes – were imported into and classified using Nvivo® 10 (QSR International, Burlington, MA, USA). Two investigators (AMAD and KB) first open-coded 5 source documents in each category (interviews vs. observations) to derive an initial or broad code list and then, together, refined this list through consensus discussion and comparison of codes back to original content. The broad codes were distinguished by perspective (i.e. patients vs. caregivers vs. clinicians); thus allowing an inductive process of general themes to develop. The final code book also included deductive codes related to the CuCoM, [3] the Burden of Treatment Theory, [5] and the Theory of Patient Capacity [4]. They tested five more cases to identify any additional themes (inductive) and measure, validate and achieve agreeability, and conformity of coding (i.e. consistency). When a coder identified a new theme while coding subsequent source documents, both coders met to discuss inclusion into the code book (synthesizing). After coding, they synthesized information and compared similar and variant themes between and across cases (theorizing), and then compared these themes with elements from the theories supporting the MDM model of care listed above (recontextualizing) (Fig. 2). During these various stages, we maintained a continuous member checking and calibrating to ensure accuracy, credibility, and trustworthiness.

## Results

The participating patients and caregivers included male and female participants (%14 female participants), with ages ranging between 23 and 82 years old. The length of diagnosis varied between participants from months to over 20 years. The socioeconomic status of patients is summarized in Table 1.

**Table 1:** Summary of sociodemographic variables of the interviewed participating patients

Sociodemographic variable	Number (***N*** = 28)
**Sex**	
Male	24
Female	4
Other	0
**Age (range)**	
18–29	0
30–49	9
50–69	17
≥ 70	2
**Marital Status**	
Single, never married	9
Married	10
Widowed	1
Divorced	3
Partnered	3
Separated	2
**Race**	
Caucasian	26
Black	2
Hispanic	0
Other	0
**Employment Status**	
Employed (active)	21
Not Employed/Disable	2
Retired	3
Unknown/Non-disclosed	2
**Living situation**	
With someone else	14
Alone	14
**Income (range)**	
≤ $25,000	3
> 25,000-49,999	3
> 50,000-74,999	2
> $75,000	9
Not disclosed	11

**Fig. 3:**
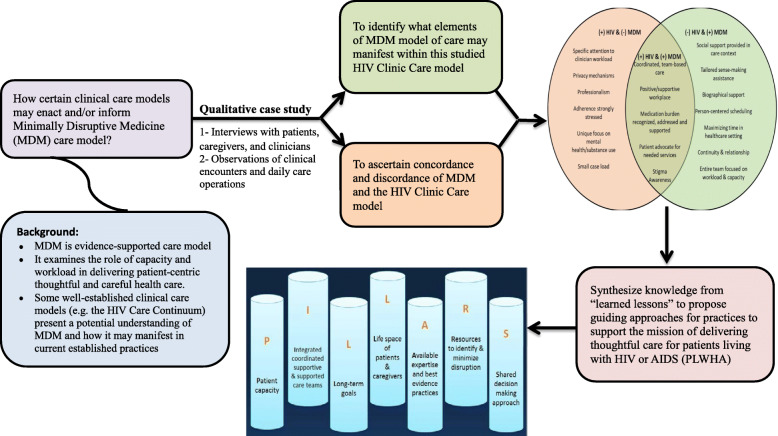
Analytic framework and diagram summarizing the study flow from hypothesis to results and findings

The participating clinicians included male and female clinicians (57% female clinicians). Interviews lengths ranged between 17 and 92 min. Observations of clinical encounters varied between 7 and 48 min. Observations of clinical and operational encounters ranged between 2 and 6 h on various days through the study period. The final dataset was comprised of various source documents from interviews with 28 patients, 5 caregivers, and 14 clinicians, as well as field notes from the researchers’ observations. In addition, two patients declined to participate while four agreed to participate and be interviewed; however, arranged interviews were not completed. The recruitment process and flowchart are summarized in Supplementary Figure 1. An analytic framework and diagram summarizing the study flow and findings is shown in Fig. 3.

The care provided in this clinic mostly supported patient capacity (with some patients receiving minimal social support and limited assistance with reframing their biography) and minimized workload (with some patients challenged by the clinic hours of operation); thus, aligning with the framework of MDM care model. The findings also presented unique elements to MDM, including attention to life-work harmony and work hours load. The main findings in Fig. 2 illustrate clinic features that were unique to the HIV clinic and not necessarily accounted for in MDM [(+)HIV Clinic model & (−)MDM]; the features of the clinic that were consistent with the HIV clinic and MDM [(+)HIV Clinic model & (+)MDM]; and those that are described in MDM, but were not consistent with what was seen in the HIV clinic [(−)HIV Clinic model & (+)MDM]; Table 2 describes these findings using MDM’s frameworks for workload and capacity with the following major themes that stood out in the data.

**Table 2:** Overlap between MDM Model of Care and HIV Clinic Care

		(+)^**α**^ HIV Clinic model & (−)^**β**^ MDM	(+) HIV Clinic model & (+) MDM	(−) HIV Clinic model & (+) MDM
**Workload**	**Sense-making**		• Patient education on purposes for medication/adherence to medication	• Tailored sense-making support
	**Enrolling/** **Planning**		• Coordinated, team-based care	• Coaching to build patient capacity for self-care
	**Enacting Work**	• Adherence stressed in all sessions due to dire consequences of non-adherence	• Coordinated, team-based care • Medication burden recognized and supported	• Person-centered scheduling for all care*
	**Appraisal**		• Consistent feedback regarding viral load/success of treatment plan	
**Capacity**	**Biography/living life**	• Unique focus on mental health/substance abuse • Privacy mechanisms		• Support during biographical disruption from illness
	**Resources**		• Advocate for additional services	
	**Environment**	• Professionalism, Trust	• Positive Healthcare Environment • Coordinated, team-based care • Continuity of coordinated care	• Entire team focused on workload/capacity
	**Work**	• Team members co-location	• Additional services (such as home health) arranged for high-need cases	• Coaching to build patient capacity for self-care
	**Social**		• Social support assessed by a social worker • Stigma minimized	• Social support system understood by all clinicians • Social network support offered as part of care (e.g., patient groups, community resources)
**Other**		• Attention to clinician workload** • Manageable case load		

^α^ (+) = present in

^β^ (−) = absent from

* Although this appeared in the HIV clinic, it was recognizably inconsistent and varied between staff members

** Recently, the work of MDM has recognized that understanding clinicians’ capacity and workload are also essential components of delivering a minimally disruptive care; thus, there is a need to assess and address that

Table 3 summarizes various participants accounts from interviews and/or clinical encounters that relate to the identified major theme in Table 2.

**Table 3:** Sections of findings supported by various accounts of participants during semi-guided interviews or clinical encounters

**Section I: (+) HIV Clinic & (+) MDM (Areas of overlap)**	
a) **Care continuity through coordinated teamwork**	1. ***Patient:*** *So this is one thing we really appreciate is the team approach to everything. So, you know, we know that first we’re going to meet with the nurse and go through all of our stuff, and then we know, you know, we’re going to meet with doctor and then meet with the pharmacist. So I mean, we appreciate that … that it feels like total care.* **(Female; 40–49 years old)** 2. ***Clinician:*** *I think that it’s … that team approach is good for patients. Rather than saying—well now I want you to go see a pharmacists down in the pharmacy, and they wouldn’t go. Had we been able to get their shots right here, they’d get them.*
b) **Social lives and stigma consideration**	1. ***Patient:*** *This disease is so much more mental than it is physical, sometimes. That’s where the difference they have made, because I’ve never felt like a pariah or like, you know, the outside world view of HIV and AIDS, And you never, ever, ever felt like that in these rooms by anybody I’ve talked to, been next too, got blood from, whatever; I’ve never felt that a smidge. The stigma...Yeah, no stigma at all from when you enter this building to when you walk out, and that’s huge, because I’d be the guy that wouldn’t come back if I felt that. That’s what makes it a safe place.* **(Male; 50–59 years old)** 2. **Clinician:** *normalizing that experience with HIV is a huge thing of what we do here … and … we can only do so much here in the clinic … we can at least make this a safe place for it. Changing the whole attitude towards HIV elsewhere is … can be a life-long task … until we do it … yeah”*
**Section II: (−) HIV Clinic & (+) MDM (Present in MDM, but missing in the HIV clinic)**	
a) **Understanding life’s technicalities is not the same as biographical support**	1. ***Patient:*** *So if I’m only going to live a couple weeks, I don’t care. If I’m going to live for a year, I don’t really care (laughs). But … if I’m going to live longer...I need to figure out how to cope with it.* **(Male; 70–79 years old)** ***Patient’s caregiver:*** *And that’s why I think, maybe, the social workers could*. *. .they did. . I mean they have offered. We just weren’t ready last time.* 2. ***Patient:*** *I lost everything. I lost my house. I lost of couple of family. I lost some friends. You know. .. I lost my landlord; she was a good person. She treated me like I was her son. You know, you miss that. You know, I mean they’re all back home. I had a home, you know. I miss so much back home, you know.* ***Interviewer:*** *Do you discuss these things here when you come? Do you talk to your clinician about that?* ***Patient:*** *No, no.* **(Male; 40–49 years old)** 3. ***Clinician:*** *I mean, unless they bring it up, it’s like. .. so I guess I ask—how are you doing. .. but that’s pretty open. And some people will share something, but I feel like the clinic visits are so long. .. . that some people just don’t. .. . they just want to see you and get moving onto the next person because they already know they’re going to be here a long time.*
b) **Danger in delegation of social issues**	1. ***Clinician:*** *Sometimes it turns into … some of our folks that are in case management … um … it almost feels like … eh … dependent relationship” “for them to be able to function more independently is very difficult for them, and they really needed to have that person (social worker) to help them navigate through that complex world of medical appointments and* etc. 2. ***Clinician:*** *That is what I do and typically for the patient there is a couple of reschedules before they actually see a doctor and they feel bad calling … So, it is they start to feel like “I need to have that person there to help or I will just run into that barrier … and then they get frustrated and kinda give up. It can be challenging as a case manager to try not have that real dependence...dependency build up with the patient so we can they can still feel that, yes, they can do, and that I am here as a support”*
c) **Needless work and needing social support**	1. ***Patient:*** *So, I’ve been having a lot of appointments here at the clinic, and it’s just that I don’t want to keep coming back if it’s not going to accomplish something. These visits just to see how things are going, you know, they could find that out with a phone call.***(Male; 60–69 years old)** 2. ***Clinician*** *“One of the barriers of the team approach is that, I think … we all are important, we all wanna see the patients … .but sometimes I think, it gets overlapped, and then … eh … we even are providing sometimes too much to some patient that maybe the needs aren’t there”* 3. ***Patient:*** *[Another clinic] had just resources on the walls available, like not necessarily specific to the clinic, even just social support, which I feel, sure I get support for my own here, but outside of here, I don’t feel like there’s support or resources to get that support.* **(Male; 50–59 years old)**
**Section III: (+) HIV & (−) MDM (Present in the HIV clinic, worth incorporating into MDM)**	
a) **Enabling factors to work-life harmony**	1. ***Clinician:*** *I’m quite happy with my work-life balance. The clinic is typically a busier day, but not to a point that I’ve ever had an issue.* 2. ***Clinician:*** *So in that area in XXXX, they had way more visits, and the pharmacists couldn’t see everybody cuz they only had one pharmacist for the unit. ..*. *the one unit or area. So the pharmacy visits are typically tiered or selected by the providers, so that’s a little bit different, whereas here, a pharmacist is seeing every patient, reviewing every patient, and that’s beneficial.*
b) **Privacy and professionalism**	1. ***Patient:*** *So while we were waiting for our appointment here, we saw two different people in XXXX, and I can tell you the quality of care that we’ve gotten here ten times surpasses the quality of care we got there.* ***Interviewer:*** *So I want to ask you—what is different. .. what did you view different between here (this clinic) and there (the previous clinic you attended)?* ***Patient’s caregiver:*** *Um, I mean straight up, out of the two, I would say honestly its level of knowledge. I mean, hands down. You know, it’s truly expertise, know-how; they just really...* ***Patient:*** *And professionalism, I would say.* **(Female; 40–49 years old)**

The following further details our findings of elements from Table 3 accompanied by our observations:

I. Areas of Overlap

- Care continuity through coordinated teamwork

Patients and clinicians noted how multidisciplinary and coordinated care offered often by the same team member accomplished care continuity, and how this feature minimized treatment workload for patients. (Table 3: section I.a.1).

We observed how this coordinate care structure provided the care teams the “full picture” that each team member contributes; thus, providing a truly person-centric, expert-provided care that fits a patient’s context of life, goals, and preferences.

b Social lives and stigma consideration

Positive social support is noted as a key component of patient capacity for self-care. Patients described the role that social support and stigma related to the HIV infection plays in their lives. The clinic team made deliberate efforts to incorporate this knowledge into their care (Table 3: section I.b.1).

In our observation, we noted how clinicians designated first-visit-to-establish-care as an opportunity to mainly introduce team members, and discuss and help patients understand living with HIV, with emphasis on addressing social life concerns as well as the role of stigma as a barrier to various aspects of personal and professional aspects, and therefore, its impact on their social realm. This was brought up or discussed again in various visits with patients with established care.

II. Present in MDM, but missing in the HIV clinic

- Understanding life’s technicalities is not the same as biographical support

Chronic illnesses, and their acute exacerbations or associated complications, disrupt patients’ biographies (i.e., who they are and how able they are of authoring their stories; biographical support reflects any type of support that enhances that person’s life with all of its aspects to live, approach, and/or shape it up). HIV infection and the conditions that sometime accompany it can be quite disruptive. Some participants were in the throes of biographical disruption. These patients expressed active distress. However, this was not always apparent in interactions with the healthcare team, and clinicians did not always inquire about these issues, as one patient noted (Table 3: section II.a.1) and another patient also accounted (Table 3: section II.a.2).

In fact, clinicians seemed to think patients would bring these life issues up in their visits if there was something to talk about, whereas patients didn’t feel ready to bring these issues up themselves – it was significant emotional work to get the nerve to do just that, leaving a vacuum of important issues unaddressed (Table 3: section II.a.3).

To the extent that patients were asked about these issues during their visits, it was primarily to tailor the medication regimen (e.g., shift work times) or to target practical support (e.g., transportation assistance) to make sure that patients adhere to the plans and appointments.

b Danger in delegation of social issues

The social worker was the team member most aware of patient situations, often giving other clinicians a summary of social and physical living conditions prior to their visit when challenges were present. Social workers were trained to assess patients coping abilities and financial resources, and were responsible for making connections with community resources. New patients met with the social worker initially to assess barriers to adherence and access to care. Social workers typically served as case managers for patients experiencing certain barriers, including living well below the state-defined poverty line to support managing living conditions (e.g. housing affordability, access to care and medication, heat bills). Otherwise, patients saw the social worker once a year or when social issues emerged prompting clinician referral. Given patients’ reluctance to mention such issues, social work may have been underused, overused, or under-integrated (Table 3: section II.b.1).

c Needless work and needing social support

Because of the intentional focus of the clinic on patients’ adherence, the clinic sought to ensure patients had follow-up visits every three months. These visits were often disruptive for patients because of travel to the center and the clinic’s set up of hours-long, afternoon-only appointments. When patients saw the value of the visits, they didn’t mind, but in cases where there was little or no perceived value, they felt this was not used properly (Table 3: section II.c.1, 2).

Also, while patients felt supported at the clinic, their care did not typically include facilitating access to social support, such as in-person or online patient communities (Table 3: section II.c.3)

III. Present in the HIV clinic, worth incorporating into MDM

- Enabling factors to work-life harmony

The ability to provide successful team-based care may have been due in part to two other, more systemic factors noted in the data: attention to clinician workload and a reasonable clinic volume (Table 2: section III.a.1, 2).

Although original work on MDM did not specifically emphasize an understanding of potential burden on clinicians and healthcare systems; recently, the work of MDM has recognized that understanding clinicians and health systems’ capacity and workload are also essential components of delivering minimally disruptive care [2, 26, 27].

b Privacy and professionalism

Patients noticed how the HIV clinic, operating within an infectious disease clinic, protected their privacy. For example, a patient noted how they had switched from calling patients by name in the waiting room to discretely paging them. The team’s professionalism contributed to patients’ trust in them and in the care they offered (Table 3: section III.b.1).

## Discussion

### Principal findings

This study observed the care presented at a specialized HIV-care clinic to understand which elements of this care were concordant or discordant with MDM. With a few exceptions, the HIV clinic operated in a manner closely aligned to the MDM model of care. This care mostly supported patient capacity (with some patients receiving minimal social support and limited assistance with reframing their biography) and minimized workload (with some patients challenged by the clinic hours of operation).

### Limitations and strengths

Our study based on interviews and observations at a single and mature HIV clinic—one following a well-rehearsed approach typical of clinics funded by the Federal Ryan White grants, and the US federal 340B pharmacy program —may offer only a “best-case” scenario. Reports from people available for interview at the clinic may not represent well the views of those who had difficulty attending appointments, thus it may have missed features that failed to support people with limited capacity. Our participating patient sample size, however, provides a comparable socio-demographic representation of the general population seen within the HIV clinic. Additional strengths of our approach also derive from the implementation of a rigorous, inductive, and collaborative approach, using two coders, a shared and mutually established codebook, and regular member check meetings during the analysis process. We expect that our findings will support policy makers, stakeholder, and users of various clinical settings to determine the potential pertinence, applicability, and usability of our findings to their settings.

### Relationship to other literature

The team-based care model observed in the HIV clinic we studied is the least common model of care [28]. Few studies have examined the extent to which existing care models align with the concepts of MDM [26]; where they have, with the exception of this report, they have found little alignment [29]. From the perspective of patients and healthcare professionals, the examined model of care seems to overcome specific issues identified as challenging to patients’ workload-capacity balance [9]. For example, past research has identified clinic-level factors preventing adherence and retention in care such as trouble getting appointments, lack of communication amongst clinicians, uncaring clinical teams, and inability to customize patient care plans to fit their lives [16, 19, 30, 31]. These factors can also reduce patient capacity [4].

Our observations indicate that clinic procedures sought to minimize burden in gaining access to appointments, in communicating with and amongst team members, and in working collaboratively with patients to customize treatment plans to fit their lives. This general observation, however, needs to be realized in the care of a range of patients. For example, the clinic made efforts to integrate in one afternoon a range of highly coordinated clinic visits with members of its multidisciplinary care team; for some patients, including those who travel long distances to the clinic, this seemed respectful of their limited time, energy and attention; for others it was exhausting. This highlights the need to tailor care acknowledging between-patient differences in their workload-capacity balance [8].

### Researcher’s reflexivity

Early on during the interviews, we witnessed a general enthusiasm in participating in research from the patients and their caregivers. On the other hand, we also observed the factors that may have limited participation (or the extent of it), especially regarding patients living in smaller communities where social stigma continues to exist. We heard tender stories that ranged from dealing with a lack of public understanding of living with HIV and the unjust social stigma. Additionally, we heard powerful stories and reactions from patients and clinicians during our interviews; in one time, a clinician spoke with teary eyes stating “They are people like us. They deserve to be treated as such. This stigma they experience breaks my heart”.

While such accounts triggered emotions within us, it was very important to listen to them and sentiments from all participants, while maintaining presence, respect, and separation of any biases that could hinder our objective and observatory process. Discussing these observations within our team helped in maintaining this approach and also how we understood the progress of findings from this case study. Our team consists of individuals with different backgrounds in clinical and research knowledge, expertise, and experiences. Such enriching background made for a positive collaboration and synergy to understand how MDM and this clinic care model manifested while maintaining objectivity. None of our interviewing team members had established connections with any of participants.

### The PILLARS-- a proposed guiding typology for clinical practices

**Fig. 4:**
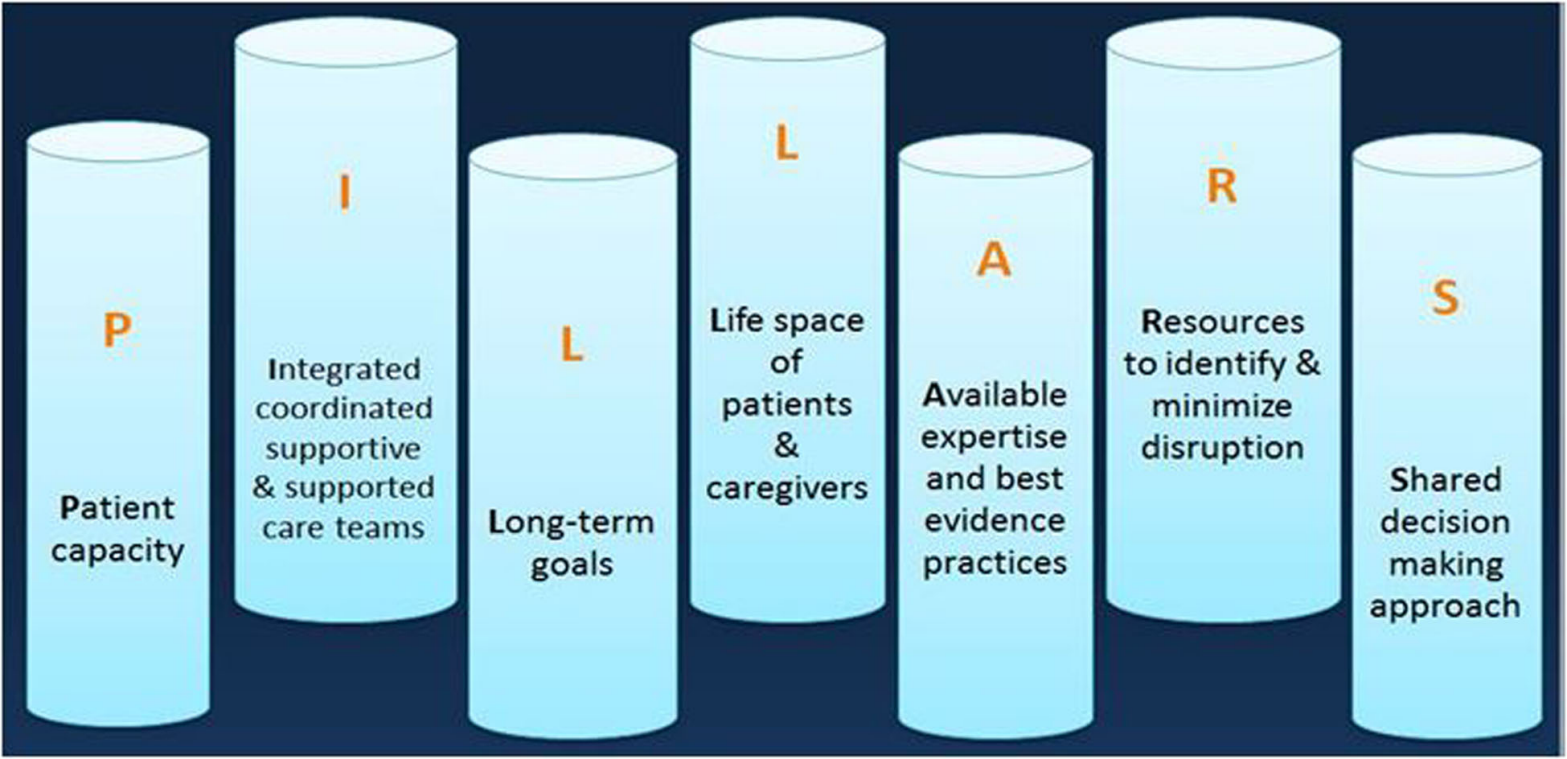
PILLARS of MDM in designing systems for care

Based upon our findings of both concordant and discordant aspects of the HIV Clinic with the MDM Model of Care, we propose a potentially helpful typology of the PILLARS. This healthcare would consider; what impacts Patient capacity as part of treatment strategies; building Integrated and coordinated care teams that deliver supporting care while nurturing a culture of supportive, teamwork environment; the Long-term goals and preferences of the patients; the Lifespace and context of life for the patients (and their caregivers, when applicable); Available and existing expert opinions coupled with the best available evidence to support their practices; identifying and employing Resources that support minimizing care disruption for patients; and approaches of Shared decision making culture to provide this care, as shown in Fig. 4. This will provide a guiding approach for clinic administrators and policy makers to consider when exploring best approaches to designing or re-designing healthcare that are in alignment with MDM.

### Implications for practice and research

Despite a decade of conceptual and theoretical work to describe it, measure it, and support it in practice with conversation aids, [1, 3–5, 32–35] MDM has never been applied in full scale to the care of patients. This case study explores the extent to which a practice model driven by the critical need to assist patients in accessing care and adhering to treatment in order to assure reduced patient mortality and increased quality of life has organically developed characteristics of MDM, i.e., ensuring patient workload-capacity balance, reducing treatment burden, offering holistic capacity support [36, 37]. This further supports the practical value of MDM and contributes toward validating its tenets and aligns with other findings that call for care that emphasizes on being minimally disruptive [37]. The majority of the elements found in the program did align with MDM. However, an opportunity exists for practices to ensure biographical and social support, as well as to tailor care to each patient’s unique needs to reduce treatment burden [33]. Identifying these gaps was made possible by applying the theoretical frameworks that support MDM to the analysis of clinical services. Furthermore, the PILLARS typology describes a helpful actionable set of practice components in alignment with MDM that clinics can use to guide their design of services to meet the needs of patients living with chronic conditions with consideration to the capacity of the healthcare teams and staff.

Research is limited or non-existent regarding the extent to which the team-based care model for HIV and MDM respectfully impact patient outcomes [28]. Patient reports’ in this study as well as in previous research highlight that a focus on minimally disruptive and maximally supportive care is more patient-centered, and to the extent that the clinic studied here was in alignment with MDM, hope that future care can become implemented. Future HIV research as well as research on chronic illness should focus on *how* to implement MDM team-based care with fidelity to MDM principles, and *what* impact this care has on patients’ outcomes, including outcomes such as mortality and quality of life. It is also important to be mindful of the intrinsic differences and challenges that healthcare provided for chronic conditions like HIV manifests uniquely, including understanding of social stigma and importance of adherence. The PILLARS typology suggested here offers a useful scaffold for future practice interventions (i.e., activities of appropriate focus and exploration), and the template for intervention description and replication (TIDieR) checklist and guide reporting criteria should be referenced to ensure proper reporting of intervention components [38].

## Conclusions

This case study found that this HIV clinic has evolved in a manner that incorporates many of the tenets of MDM, contributing to its validation. We also found that the theoretical frameworks underlying MDM can help identify and interpret gaps in care for patients with chronic conditions. These findings may support the design and implementation of care that is both minimally disruptive and maximally supportive. In addition to extending these observations to other clinics and other chronic conditions in practice, the extent to which this transition in care toward MDM impacts patient-important outcomes requires further testing.

## Supplementary Information

**Additional file 1.**

**Additional file 2.**

## Data Availability

The datasets generated and/or analysed during the current study are not publicly available due to the studied condition’s sensitivity and strict confidentiality assured for participants but are general inquiries about the data is possible to the corresponding author on reasonable request and under limited circumstances and conditions.

## Abbreviations

MDM: Minimally Disruptive Medicine
HIV: Human Immunodeficiency Virus
PLWHA: Patients living with HIV or AIDS
CuCoM: Cumulative Complexity Model
SRQR: Standards for Reporting Qualitative Research
IRB: Institutional Review Board
PGY: Postgraduate Year
