# Supplementary material for: Minimally disruptive medicine (MDM) in clinical practice: a qualitative case study of the human immunodeficiency virus (HIV) clinic care model
Source: BMC Health Serv Res. 2021 Jan 6;21:24. doi: 10.1186/s12913-020-06010-x (PMC7788961; doi:10.1186/s12913-020-06010-x)
Supplement: Supplementary file 1 — Additional file 1. [file 12913_2020_6010_MOESM1_ESM.docx]

**Guided Questions (Patients Interviews)**

1. How is/was your visit?
2. Can you describe a typical visit of yours to this clinic; what goes into it?
3. As you come here to the clinic for your appointments today, did you need to change your daily schedule around?

- **Probe1:** Coming to your appointments, do you feel that you need to work things around your life to make that happen?
- **Probe2:** Does it disrupt your life?
- If yes: **Probe1:** How so? **Probe 2:** In what ways?
- If no: **Probe2:** So, it fits really well into your life and your schedule?

1. In general, and for any visit, how does the care you receive here affect your daily life and schedule outside the clinic?
   - **Probe1:** Caring for your health and condition, what efforts or work that adds to your daily life?
   - **Probe2:** Setting appointments, taking medications, someone to drive you if you are not feeling well, missing events…. etc.
2. When you come here to the clinic, say like today, do you discuss that (Q3) with your clinicians (anyone)?

- **Probe1:** Do you tell your any of the clinicians or staff about if coming to your appointment changes or adds efforts or time to your day, if any?
- **Probe2:** Does anyone of the staff here ask if coming to your appointment changes or adds efforts or time to your day, if any?
- **Probe3:** If answered yes or no -- what are these things?

1. Having this discussion and talking to the staff about your care here, how does that help you caring for yourself, if any?
2. How does that discussion help you with making decisions to deal with your daily life activities or schedule?

- **Probe1:** Say, if you discussed how coming to clinic for your appointments or any type of care you get here, does that affect taking decisions about your daily life? Schedule for your day or week? When to take your medications?

1. How do you view the care you receive at this clinic any different, if so, from anywhere else where you get your care?

- **Probe1:** Compared to any prior clinic you have been to, whether for HIV care or for any other health issue (PCP or specialty), how is this clinic different, if so?

1. While receiving this care and caring for yourself, are there any things that you don’t fit in your life?

- **Probe1:** Do you feel that anything might work differently to help you better care for your life?

1. Are there things that you’d like to be doing otherwise?

- **Probe1:** How would you spend the time if you weren’t doing things to manage your health or coming to appointments?
- **Probe2:** What this clinic can be doing to make you able to do these things?

**Guided Questions (Staff Interviews)**

1. How do you approach delivering care to patients at this clinic; what goes into it?
2. Are there any aspects in their lives you like to ask about regarding their care?
3. Do you see opportunities to discuss with patients when it comes to taking care of themselves, daily outside the clinic? (Prop: e.g. simple daily tasks, social life, going outdoors…etc.)
4. If that happens, how does that discussion go?
5. How do you see the care provided here affecting your patients’ daily life outside the clinic?
6. Through caring for your patients in this specialty clinic, what do you view in this care that is any different from other care that might be given to them somewhere else?
7. What obstacles patients might face while receiving their care here, if any?
8. What is your opinion on the system built to care for patients through this clinic? (Prop: How does it work for the sake of the patients?)
9. What challenges, if any present, do you face caring for the patients in this specialty clinic? (e.g. Mayo Clinic’s geographic location, paper-work, relationships, research-related)
10. How caring for your patients here affects your life, if at all?
