# Supplementary figures and images for: Minimally disruptive medicine (MDM) in clinical practice: a qualitative case study of the human immunodeficiency virus (HIV) clinic care model
Source: BMC Health Serv Res. 2021 Jan 6;21:24. doi: 10.1186/s12913-020-06010-x (PMC7788961; doi:10.1186/s12913-020-06010-x)

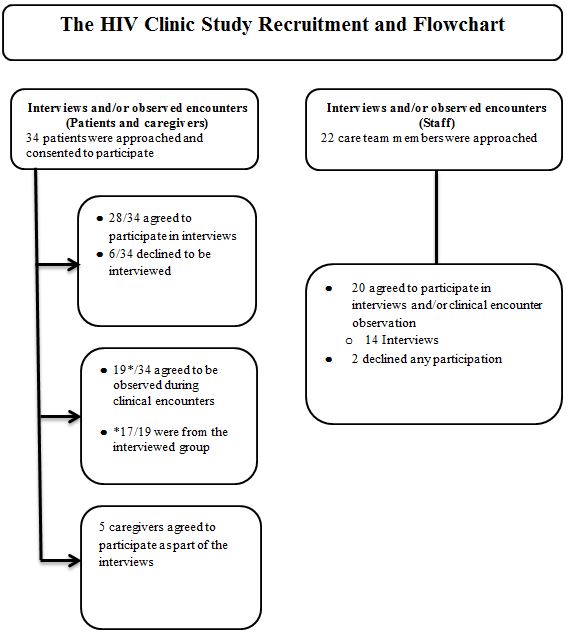

Supplement: Supplementary file 2 — Additional file 2. [file 12913_2020_6010_MOESM2_ESM.jpg]
